# 

**Published:** 1957-07

**Authors:** 


### MEDICAL JOURNAL OF THE SOUTH-WEST

*(Incorporating the Bristol Medico-Chirurgical Journal)*

Established 1883

VOL. 72 (iii) | July, 1957 | NO. 265

### *Contents*

| | | Page |
|---|---|---|
| A TOUR OF THE SOUTH-WESTERN PACIFIC | *Mr. A. L. Eyre-Brook* | 75 |
| THE PREVENTION OF ACUTE RHEUMATISM | *Prof. C. Bruce Perry* | 86 |
| HEAD INJURIES . . . . . | *Mr. G. L. Alexander* | 91 |
| BRISTOL AND BORDEAUX . . . . | *Dr. J. A. Cosh* | 98 |
| SARCOIDOSIS TREATED WITH CORTISONE | *Clinical Pathological Conference* | 101 |
| NOTES AND NEWS . . . . . . . | | 106 |
| APPOINTMENTS . . . . . . . . | | 107 |

*PUBLISHED QUARTERLY*

*ANNUAL SUBSCRIPTION* 16/- *POST FREE*

PUBLISHED UNDER THE AUSPICES OF THE
BRISTOL MEDICO-CHIRURGICAL SOCIETY

### *Editorial Committee*

Dr. J. E. Cates, Department of Medicine, Bristol Royal Infirmary. *Editor.*

Dr. N. J. Brown, Southmead Hospital, Bristol. *Assistant Editor.*

Dr. J. Macrae, Ham Green Hospital.

Dr. R. C. Wofinden, Central Clinic, Bristol.

Mr. A. E. S. Roberts, Medical Library, University of Bristol. *Secretary.*

### *Local Correspondents*

*Bath* . . Mr. A. D. Bateman, 3 The Circus, Bath.

*Exeter* . . Dr. L. N. Jackson, Mount Jocelyn, Crediton, Devon.

*Gloucester* . Dr. R. F. Jarrett, 9 College Green, Gloucester.

*Plymouth* . Dr. C. R. Croft, Lexden, Hartley Av., Plymouth.

*Taunton* . Dr. M. McAlley, 1 The Crescent, Taunton.

*Torquay* . Dr. A. Robinson Thomas, 20 Devon Square, Newton Abbot, Devon.

*Truro* . . Dr. F. D. M. Hocking, St. Andrews, Perranporth, Cornwall.

*Yeovil* . . Mr. J. A. Vere Nicoll, Knapp House, Yeovil, Somerset.

### NOTICE TO CONTRIBUTORS

Original articles are invited, provided they have not been published elsewhere. Notes of forthcoming events, meetings held, degrees conferred, staff appointments, and other local news are welcomed. The editorial committee reserves the right to make literary corrections.

Illustrations should always be supplied ready for reproduction. All re-touchings or other operations required will be charged to the author. Line drawings should be drawn in Indian ink. We regret that we must ask authors to pay for making blocks, if this is necessary.

J. W. ARROWSMITH LTD., WINTERSTOKE ROAD,
BRISTOL.

### *Notes and News*

The Board of the South-West Faculty of the College of General Practitioners has decided to institute an annual lecture in memory of the late Dr. A. H. Gale. It will be given each year prior to the Annual Meeting of the Faculty. The inaugural lecture will be given at Torquay on Sunday, 13th October, by Dr. R. S. Hope-Simpson, of Cirencester, on "Opportunities and Pitfalls of Research in General Practice".

### UNIVERSITY OF BRISTOL

*M.D.*

Sanerkin, N. G.

*M.B., Ch.B.*

WITH SECOND-CLASS HONOURS

Hockin, Janette A.
Shortman, S. C. J.

Pass

Akinsete, F. I.

Betteridge, T. J.

Bland, Pauline M.

Bowden, D. E.

Brewer, C. C. L.

Burland, W. L.

Counsell, G. F.

Daniels, S. M.

Dansie, Norma St. C.

Douglas, H. G. K.

Dowell, Bethia M. (*with Distinction in Public Health*).

English, J. M.

Faint, Sheila A.

Falkner, C. G.

Faulkner, D.

Fraser, I. D.

Fung, Joyce

Innes, D. M.

Jenkins, C. W.

King, Audrey

Koney, J. O.

Manley, G. W.

Moffatt, Jean (*with Distinction in Public Health*).

Morgan, A.

Onubogu, H. O. N.

Pengelly, D. C.

Rackham, K. T.

Robertson, D. A. S.

Rose, A. L.

Shaw, J. F.

Sheard, M. E.

Stock, P. R.

Sutton, G. B.

Taylor, Margaret

Thompson, I

Weeks, D. C.

Wotherspoon, H. G.

Curtis, G. T.

Millen, H. E.

Prescot, R. E.

Stamper, J. R.

Zutshi, D. W.

### EXAMINATION RESULTS

*M.R.C.P.*: D. R. Coles.
*L.R.C.P., M.R.C.S.*: R. J. A. Brown.

### PRIZES

*Foyle Prize for* 1957: G. Neale.
*Edward Fawcett Memorial Prize for* 1957: G. Neale.

### Appointments

### UNITED BRISTOL HOSPITALS

### MARCH TO JUNE, 1957

| Name | Appointment | Formerly |
|---|---|---|
| MacQueen, I. G., M.B., B.S., F.R.C.S. | Registrar in Orthopaedic Surgery. | Residential Surgical Officer, Tunbridge Wells Group. |
| Epszteyn, Miss L., M.B., Ch.B., D.R.C.O.G. | Registrar in Obstetrics, and Gynaecology. | Registrar, Perth Royal Infirmary. |
| Doyle, F. H., M.B., Ch.B., D.M.R.D. | Senior Registrar i n Radiodiagnosis. | Registrar in U.B.H. |
| Burley, M. A., L.D.S., (Edin.) | Registrar in Orthodontics. | Dental Officer, Colonial Service, Hong Kong. |

Dr. H. M. Leather, M.B., Ch.B., M.R.C.P., has resumed his appointment as Senior Registrar in Medicine on completion of his appointment as First Assistant in Medicine at Makerere College, Kampala, Uganda.

Dr. W. A. W. Dutton, M.B., M.R.C.O.G., has been appointed Tutor in Obstetrics and Gynaecology, Queen's University, Kingston, Ontario.

### SOUTH WESTERN REGIONAL HOSPITAL BOARD

### MAY TO JUNE, 1957

### BRISTOL

| Name | Appointment | Formerly |
|---|---|---|
| Bennett, Mrs. M. O. M.D., Obst., (Lond.), Ch.M. (Brist.), M.R.C.O.G., F.R.C.S. (Eng.). | Consultant Obstetrician and Gynaecologist, Southmead Hospital. | Clinical Assistant in Obstetrics, U.B.H. |
| Crossley, John, M.B., B.Chir. (Cantab.), F.R.C.S. (Eng.), M.R.C.O.G. | Consultant Obstetrician and Gynaecologist, Southmead Hospital. | Senior Registrar, St. Thomas's and Lambeth Hospitals. |
| Willis, Audrey S., B.A., (Cantab)., M.B., B.Chir. M.R.C.P. | Senior Medical Registrar, Frenchay Hospital. | Registrar in Department of Experimental Medicine, Cambridge. |
| Solling, M. C. R., M.B., B.S. (Sydney). | Surgical Registrar, Cossham-Frenchay Group of Hospitals. | S.H.O. in General & Orthopaedic Sgy., North Middlesex Hospital. |

### NORTH GLOUCESTERSHIRE

| Name | Appointment | Formerly |
|---|---|---|
| Ferguson, J., M.B., B.S. (Melbourne). | Medical Registrar, Gloucestershire Royal Hospital. | Locum tenens appt. at St. Matthew's Hospital, London. |
| Greenwood, R. K., M.B., B.Chir. (Cantab.), F.R.C.S. (Eng.). | Surgical Registrar, Gloucestershire Royal Hospital. | Resident Surgical Officer, Royal Devon and Exeter Hospital. |
| Hanna, F. A., M.B., B.Ch., B.A.O., D.M.R.T., (Brist.). | Consultant Radiotherapist, North Gloucestershire Clinical Area. | S.H.M.O., North Gloucestershire Clinical Area. |

### *BATH*

| *Name* | *Appointment* | *Formerly* |
|---|---|---|
| Bazeley, R. W., M.B., B.S. (Lond.). | Re-appointed for further year as Clinical Assistant in General Medicine, Bath Group of Hospitals. | In general practice, Radstock. |
| Ellison, D. J., M.B., Ch.B., (Edin.), M.R.C.P. (Edin.). | Re-appointed for further year as Clinical Assistant in General Medicine, Bath Group of Hospitals. | In general practice, Melksham. |
| Ronn, H. H., M.B., B.S. (Lond.), M.R.C.P. (Lond.). | Re-appointed for further year as Clinical Assistant in General Medicine, Bath Group of Hospitals. | In general practice, Westbury. |
| Pinching, J., B.M., B.Ch. (Oxon.), M.R.C.P. (Lond.). | Appointed for one year as Clinical Assistant in General Medicine, Bath Group of Hospitals. | In general practice, Wells. |
| Higgins, P. J., M.B., B.Chir., (Cantab.). | Appointed for one year as Clinical Assistant in Paediatrics, Royal United Hospital, Bath. | In general practice, Westbury. |
| Lumb, G. N., M.B., B.S. (Lond.), F.R.C.S. (Eng.) | Surgical Registrar, Bath Group of Hospitals. | S.H.O. in Urology, Buchanan Hospital, St. Leonards-on-Sea. |
| Pringle, H. M., M.B., B.S. (Sydney). | Surgical Registrar, Bath Group of Hospitals. | Surgical Registrar, West Suffolk General Hospital. |

### *DEVON AND CORNWALL*

| *Name* | *Appointment* | *Formerly* |
|---|---|---|
| Trotter, J. K., M.R.C.S., L.R.C.P., D.A., F.F.A., R.C.S. | Consultant Anaesthetist, West Cornwall Clinical Area. | S.H.M.O., High Wycombe & Amersham Hospital Group. |
| Arthur, L. J. H., M.B., B.Chir. (Camb.), D. (Obst.) R.C.O.G., M.R.C.P. (Lond.) | Paediatric Registrar, South Devon and East Cornwall Hospital, Plymouth (joint appt. with the U.B.H. | Medical Registrar to the Professorial Unit, Royal Victoria Infirmary, Newcastle-upon-Tyne. |
| Barlow, D. J. H., M.B., B.Ch. (Witwatersrand). | Registrar in Psychiatry, Moorhaven Hospital, Ivybridge. | S.H.O., Fulbourn Hospital, near Cambridge. |
| Dhillon, B. S., M.B., B.S. (Punjab)., F.R.C.S. (Edin. and Eng.). | Senior Surgical Registrar to the Thoracic Unit, Hawkmoor Chest Hospital, Bovey Tracey. | Registrar to Neurosurgical Department, Sheffeld Royal Infirmary. |

